# Combination of lung ultrasound (a comet-tail sign) and N-terminal pro-brain natriuretic peptide in differentiating acute heart failure from chronic obstructive pulmonary disease and asthma as cause of acute dyspnea in prehospital emergency setting

**DOI:** 10.1186/cc10140

**Published:** 2011-04-14

**Authors:** Gregor Prosen, Petra Klemen, Matej Strnad, Štefek Grmec

**Affiliations:** 1 Center for Emergency Medicine, Ulica talcev 9, 2000 Maribor, Slovenia; 2 Medical Faculty, University of Maribor, Slomškov trg 15, 2000 Maribor, Slovenia; 3 Medical Faculty, University of Ljubljana, Vrazov trg 2, 1000 Ljubljana, Slovenia; 4 Faculty of Health Sciences, University of Maribor, Žitna ul. 15, 2000 Maribor, Slovenia

## Abstract

**Introduction:**

We studied the diagnostic accuracy of bedside lung ultrasound (the presence of a comet-tail sign), N-terminal pro-brain natriuretic peptide (NT-proBNP) and clinical assessment (according to the modified Boston criteria) in differentiating heart failure (HF)-related acute dyspnea from pulmonary (chronic obstructive pulmonary disease (COPD)/asthma)-related acute dyspnea in the prehospital setting.

**Methods:**

Our prospective study was performed at the Center for Emergency Medicine, Maribor, Slovenia, between July 2007 and April 2010. Two groups of patients were compared: a HF-related acute dyspnea group (*n *= 129) and a pulmonary (asthma/COPD)-related acute dyspnea group (*n *= 89). All patients underwent lung ultrasound examinations, along with basic laboratory testing, rapid NT-proBNP testing and chest X-rays.

**Results:**

The ultrasound comet-tail sign has 100% sensitivity, 95% specificity, 100% negative predictive value (NPV) and 96% positive predictive value (PPV) for the diagnosis of HF. NT-proBNP (cutoff point 1,000 pg/mL) has 92% sensitivity, 89% specificity, 86% NPV and 90% PPV. The Boston modified criteria have 85% sensitivity, 86% specificity, 80% NPV and 90% PPV. In comparing the three methods, we found significant differences between ultrasound sign and (1) NT-proBNP (*P *< 0.05) and (2) Boston modified criteria (*P *< 0.05). The combination of ultrasound sign and NT-proBNP has 100% sensitivity, 100% specificity, 100% NPV and 100% PPV. With the use of ultrasound, we can exclude HF in patients with pulmonary-related dyspnea who have positive NT-proBNP (> 1,000 pg/mL) and a history of HF.

**Conclusions:**

An ultrasound comet-tail sign alone or in combination with NT-proBNP has high diagnostic accuracy in differentiating acute HF-related from COPD/asthma-related causes of acute dyspnea in the prehospital emergency setting.

**Trial registration:**

ClinicalTrials.gov NCT01235182.

## Introduction

Acute congestive heart failure (CHF) is one of the main causes of acute dyspnea encountered in prehospital emergency settings and is associated with high morbidity and mortality [1-3]. The early and correct diagnosis presents a significant clinical challenge and is of primary importance, as misdiagnosis can result in deleterious consequences to patients [4-6]. Rapid bedside tests, especially brain natriuretic peptide (BNP) and N-terminal pro-brain natriuretic peptide (NT-proBNP), help in determining the cause of acute dyspnea in the prehospital setting [2,7]. Point-of-care bedside lung ultrasound has also become a useful method for diagnosing CHF [8]. The technique is based on the recognition and analysis of sonographic artefacts caused by the interaction of water-rich structures and air, called comet tails or B lines. When such artefacts are widely detected on anterolateral transthoracic lung scans, diffuse alveolar-interstitial syndrome can be diagnosed and the exacerbation of chronic obstructive pulmonary disease (COPD), another important cause of acute dyspnea, can be ruled out. Lichtenstein *et al. *[9] first described comet-tail signs or B lines indicating interstitial pulmonary edema, and Lichtenstein and Mezière [10] described a systematic approach to lung ultrasound. Volpicelli *et al. *[11] proposed a simplified ultrasound approach to diagnosing the alveolar-interstitial syndrome at bedside. Liteplo *et al. *[12] combined emergency thoracic ultrasound and NT-proBNP to differentiate CHF from COPD in the emergency department.

The aim of our study was to determine the diagnostic accuracy of bedside lung ultrasound (bilateral comet-tail sign or multiple vertical B lines, referred to as "lung rockets"), NT-proBNP and clinical assessment in differentiating heart failure (HF)-related acute dyspnea from pulmonary (COPD/asthma)-related acute dyspnea in the prehospital setting (that is, in the field).

## Materials and methods

This prospective cohort study was performed in the prehospital emergency setting (Center for Emergency Medicine, Maribor, Slovenia) between July 2007 and April 2010. The study was approved by the Ethical Review Board of the Ministry of Health of Slovenia. During the period of the study, 248 consecutive patients with acute dyspnea were treated by emergency teams (emergency physician, registered nurse and medical technician/driver in an ambulance or at the prehospital emergency medical center). After prehospital care, all patients were admitted (for clinical reasons and/or because they fit the study design criteria) to the University Clinical Center Maribor and followed until discharge.

**Figure 1:**
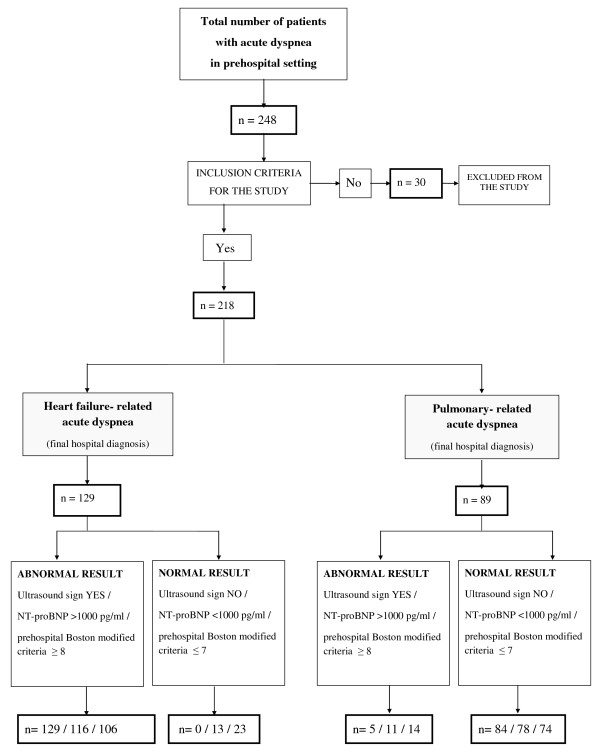
**Flow diagram illustrating recruitment, exclusion and subsequent grouping of all patients in the study**. NT-proBNP, N-terminal pro-brain natriuretic peptide.

The inclusion criterion for the study was shortness of breath as the primary complaint (defined as either the sudden onset of dyspnea without history of chronic dyspnea or an increase in the severity of chronic dyspnea). Exclusion criteria were age < 18 years, history of renal insufficiency, trauma, severe coronary ischemia (unless patient's predominant presentation was dyspnea) and other causes of dyspnea, comprising pneumonia, pulmonary embolism, carcinoma, pneumothorax, pleural effusion, intoxication (drugs), anaphylactic reactions, upper airway obstruction, bronchial stenosis and gastroesophageal reflux disorder, according to the history, clinical status and additional laboratory tests available in the prehospital setting (D-dimer, troponin, C-reactive protein). Among 248 patients, 218 met the criteria for inclusion in the study. The distribution of all patients is shown in Figure 1.

After enrollment, patients' demographic characteristics, symptoms and signs, medical histories, medication use, chest X-rays and standard blood test results (after admission to the hospital) were recorded. Our protocol for clinical assessment of HF-related acute dyspnea (the prehospital clinical assessment of HF) was designed according to the Boston criteria [6] and the Framingham criteria [13] for HF and was explained in our previous study [2] (Table 1). For additional evaluation of patients with suspected obstructive causes of dyspnea, we included criteria for clinical assessment of severe asthma [14,15] and COPD exacerbation [16] with the value of modified Boston criteria for HF being ≤ 5.

**Table 1:** Study protocol for prehospital clinical assessment of HF (modified Boston criteria)^a^

Criterion		Point value^b^
Category I: History	Rest dyspnea	4
	Orthopnea	4
	Paroxysmal nocturnal dyspnea	3
	Dyspnea while walking on level area	2
	Dyspnea while climbing	1

Category II: Physical examination	Heart rate abnormality (1 point if 91 to 110 beats/minute; 2 points if more than 110 beats/minute)	1 or 2
	Jugular venous elevation (2 points if greater than 5 cmH_2_O; 3 points if greater than 5 cmH_2_O plus hepatomegaly or edema)	2 or 3
	Lung rales (1 point if basilar; 2 points if more than basilar)	1 or 2
	Wheezing	3
	Third heart sound	3

Category III: Additional minor criteria	Hepatojugular reflux	1
	ECG changes (HLV, old AMI or nonspecific ST-T changes, arrhythmia)	1
	Night cough	1
	Murmur	1
	Without sputum and/or fever	1
	Previous AMI, arrhythmia or HF	1
	HF medications	1

^a^Boston criteria [6]. HF, heart failure; ECG, electrocardiogram; HLV, hypertrophy of the left ventricle; AMI, acute myocardial infarction. ^b^Point value: no more than 4 points allowed from each of three categories; hence the composite score (sum of the subtotal from each category) has a possible maximum of 12 points. The diagnosis of heart failure is classified as "definite" at a score 8 to 12 points, "possible" at a score 5 to 7 points and "unlikely" at a score of 4 points or less.

The final hospital diagnosis of HF-related acute dyspnea and pulmonary-related acute dyspnea (the hospital reference standard for HF and pulmonary diseases: asthma/COPD) was confirmed by cardiologists and/or intensive care physicians in the University Clinical Center Maribor using the reference standard definition for HF and pulmonary diseases in accordance with the previously cited instruments, including chest X-ray, echocardiographic examination, cardiac functional assessment (exercise test), pulmonary function test, complete blood count, biochemistry and invasive investigation or angiography [6,13-16].

According to these criteria, identification of independent predictors for final diagnosis of acute dyspnea was performed by examination of 27 variables (Table 2). Central venous pressure (CVP) in the field was assessed by the visualization of the external jugular vein, which correlates well with catheter-measured CVP [17].

**Table 2:** Univariate analysis for all demographic and clinical variables pertinent to diagnosis of acute HF or pulmonary disease (*N *= 218)^a^

Variables^b^	Pulmonary-related dyspnea (*n *= 89)	Acute HF-related dyspnea (*n *= 129)	*P *value^c^
Mean age, yr (± SD)	52.3 ± 15.3	70.9 ± 11.7	0.001
Sex, males/females (%)	176/87 (67%)	1,158/421 (73%)	0.74
Nocturnal dyspnea, Y/N	6/83	1/2	< 0.001
Orthopnea, Y/N	7/82	13/30	< 0.001
Cough, Y/N	17/41	34/95	< 0.001
Sputum production, Y/N	24/65	8/121	< 0.001
Fever, Y/N	21/68	7/122	< 0.001
Murmur, Y/N	8/81	29/100	< 0.001
Rales, Y/N	10/79	217/53	< 0.001
Wheezes, Y/N	12/3	47/82	< 0.001
Mean pulse rate, beats/min (± SD)	115.7 ± 14.1	106.3 ± 12.8	0.564
Jugular venous distension, Y/N	3/86	10/33	< 0.001
Lower extremity edema, Y/N	12/77	62/67	< 0.001
ECG-normal sinus rhythm, Y/N	210/17	55/72	< 0.001
Asthma/COPD medications, Y/N	65/11	13/116	< 0.001
HF medications, Y/N	33/56	245/46	< 0.001
Troponin T > 0.03 ng/mL, Y/N	8/81	49/80	< 0.001
Mean petCO_2_, kPa (± SD)	6.9 ± 1.6	3.6 ± 1.1	0.01
Mean NT-proBNP, pg/mL (± SD)	598.2 ± 361.8	2,263 ± 641.2	0.008
Mean SaO_2_, % (± SD)	75.7 ± 10.1	67.9 ± 12.8	0.76
Ultrasound examination-positive, Y/N	5/84	129/0	< 0.001
Previous arrhythmia, Y/N	7/82	52/77	< 0.001
Previous AMI, Y/N	7/82	15/28	< 0.001
Previous CHF, Y/N	17/72	233/49	< 0.001
Previous asthma/COPD, Y/N	169/19	9/34	< 0.001
ETI, Y/N	3/86	10/119	< 0.001
Mean modified Boston criteria score for diagnosing HF^d ^(± SD)	4.6 ± 1.2	10.9 ± 1.8	< 0.001

^a^Y, yes; N, no; petCO_2_, partial pressure of end-tidal carbon dioxide; NT-proBNP, amino terminal pro-brain natriuretic peptide; ECG, electrocardiogram; HF, heart failure; CHF, congestive heart failure; AMI, acute myocardial infarction; SaO_2_, arterial oxygen saturation; ETI, endotracheal intubation; COPD, chronic obstructive pulmonary disease. ^b^Results are presented as means ± standard deviation for normally distributed data or ratio or percentage for other variables. ^c^Univariate comparison was made using the χ^2 ^test for categorical variables and a t-test for continuous variables. For evaluation of diagnostic accuracy, patients were divided into two groups: HF-related acute dyspnea and pulmonary-related acute dyspnea (COPD/asthma). ^d^Modified Boston criteria according to Table 1 and Remes et al. [6].

During initial evaluation (before application of medicines), a 5-mL sample of blood was collected into a tube containing edetate calcium disodium for blinded measurement of NT-proBNP. The level of NT-proBNP was measured using a portable Cardiac Reader device (Roche Diagnostics, Mannheim, Germany) and recorded according to the special protocol. The test was finished within 15 minutes [2,18].

The bedside thoracic ultrasound was performed according to the protocol described by Cardinale *et al. *[8], Volpicelli *et al. *[11] and Liteplo *et al. *[12], in which eight zones of the lungs were scanned (two anterior and two lateral zones on each side of thorax). We used a portable ultrasound machine manufactured by SonoSite (SonoSite, Inc., Bothell, WA, USA). The 10 emergency physicians were included in the investigations, and they had to identify the presence or absence of three or more B lines in each of the eight zones. B lines (comet-tail signs) are hyperechoic reverberation artefacts that originate at the pleural line and extend vertically to the bottom of the screen. A positive ultrasound examination according to the definition of Cardinale *et al. *[8] and Volpicelli *et al. *[11] requires two or more positive zones bilaterally of eight zones measured. All emergency physicians who participated in our study had attended the World Interactive Network Focused on Critical UltraSound provider course. The length of the examination was always under 1 minute.

NT-proBNP measurements and ultrasound examinations were performed immediately after the arrival of the patient at the emergency department but before application of medication, thus our results were not altered by treatment. The raters who made the diagnosis (prehospital emergency physicians in the prehospital setting, internists at admission to the hospital and cardiologists and/or intensive care physicians at discharge from the hospital with the final diagnosis) were blinded to the results of NT-proBNP. In addition, the investigators of NT-proBNP did not collaborate in making the final diagnosis. On the other side, prehospital emergency physicians were not blinded to the ultrasound findings, because bedside lung ultrasound represents the routine method for assessment of acute dyspnea in our prehospital emergency unit. To avoid bias, the ultrasound findings were recorded by the emergency physicians but did not affect the diagnosis. The raters who made the diagnosis in the hospital were blinded to the findings of prehospital ultrasound. To our knowledge, no previous study has compared the diagnostic utility of ultrasound examination and NT-proBNP in a prehospital setting.

### Statistical analysis

Univariate comparisons were made by using the χ^2 ^test for categorical variables and an unpaired *t*-test for continuous variables with normal distribution (age, pulse rate, partial pressure of end-tidal carbon dioxide, NT-proBNP, arterial oxygen saturation and modified Boston criteria for HF). Odds ratios (ORs) and 95% confidence interval (CIs) were calculated to examine the risk of acute HF (adjusted using multiple logistic regression). Sensitivity, specificity, negative predictive value (NPV), positive predictive value (PPV), positive likelihood ratio (LR^+^) and negative likelihood ratio (LR^-^) were estimated for clinical assessment (based on the modified Boston criteria), NT-proBNP, ultrasound examination and a combination of ultrasound with NT-proBNP. The comparison of these four methods was done by using the χ^2 ^test with the Bonferroni correction for multiple comparisons. The area under the receiver-operating curve (AUROC) was also used to determine the diagnostic accuracy of the four methods in differentiating HF-related acute dyspnea from pulmonary-related acute dyspnea. Single areas were calculated and compared with univariate *Z*-score testing. We compared the areas under different curves using the technique proposed by Hanley and McNeil [19] and Jannuzzi *et al. *[20]. Statistical analyses were performed using SPSS software (SPSS Inc., Chicago, IL, USA). AUROC analysis was performed using Analyze-It software (Leeds, UK).

### Consent

The authors confirm that all patients gave their consent for study participation and potential publication of the study results.

## Results

During the period of the study, 248 consecutive patients with acute dyspnea were treated by emergency teams (129 patients with HF-related acute dyspnea and 89 patients with pulmonary-related acute dyspnea). Thirty patients were excluded from the study. The clinical and demographic characteristics of patients are presented in Table 2. The group of patients with acute HF was significantly older (mean ages 70.9 ± 11.7 years versus 52.3 ± 15.3 years; *P *= 0.001). The feasibility of ultrasound examination in the prehospital setting was 100%, and the duration of the examination was always less than 1 minute. For the identification of independent predictors for the final diagnosis of acute dyspnea, we examined 24 variables (variables with *P *< 0.05 on the basis of univariate analysis) in multivariate logistic regression analysis. Ten variables remained significant after analysis (Table 3). Evidently, there is big difference in ORs between ultrasound examinations (mean OR, 53.7; 95% CI, 28.6 to 83.5) and NT-proBNP (mean OR, 14.3; 95% CI, 8.1 to 29.4) and other variables. The ultrasound examination was the strongest predictor of acute HF.

**Table 3:** Multiple logistic regression analysis of factors used for differentiation between HF-related and pulmonary-related acute dyspnea in prehospital emergency setting^a^

Factor	OR (95% CI)^b^	*P *value^c^
Ultrasound examination	53.7 (28.6 to 83.5)	< 0.001
NT-proBNP	14.3 (8.1 to 29.4)	< 0.001
Orthopnea	6.9 (1.9 to 18.39	< 0.001
Rales	5.1 (1.5 to 12.8)	0.014
Troponin T	2.1 (1.3 to 4.6)	0.018
petCO_2_	7.6 (2.9 to 19.6)	< 0.001
HF medications	2.7 (1.3 to 5.1)	0.031
Asthma/COPD medications	0.12 (0.03 to 0.42)	0.028
Previous HF	7.4 (2.3 to 20.4)	< 0.001
Fever	0.17 (0.06 to 0.49)	0.017

^a^OR, odds ratio; petCO_2_, partial pressure of end-tidal carbon dioxide; NT-proBNP, amino terminal pro-brain natriuretic peptide; HF, heart failure; COPD, chronic obstructive pulmonary disease; CI, confidence interval. ^b^Univariable screening was performed on clinical, historical and biochemical variables to identify potential predictors of HF. Odds ratios for the presence of HF were generated and expressed with 95% CI. ^c^Multivariable analysis with logistic regression was used to identify potential predictor variables of a final diagnosis of HF (variables from univariate analysis with P < 0.05 for entry into model).

In Table 4, the sensitivity, specificity, PPV, NPV, LR^+^, LR^- ^and AUROC values are presented for ultrasound examinations (cutoff point: two or more positive zones bilaterally), modified Boston criteria (cutoff point: total 8 points), NT-proBNP (cutoff point: 1,000 pg/mL) and a combination of ultrasound examination with NT-proBNP. In comparing the methods, we found significant differences between ultrasound signs versus NT-proBNP (*P *< 0.05) and ultrasound signs versus modified Boston criteria (*P *< 0.05). All 11 patients for whom false-positive results were found using the NT-proBNP method had values higher than 1,000 pg/mL (mean, 1,564 ± 651.3; range, 1,200 to 2,750 pg/mL) and a history of HF. In all of these 11 patients, we confirmed the absence of comet-tail signs. With ultrasound, we can exclude HF in pulmonary-related dyspneic patients with positive NT-proBNP results and a history of HF. All five patients for whom false-positive results were found using the ultrasound method had NT-proBNP values less than 1,000 pg/mL (mean, 541.3 ± 265.1) and a history of COPD/asthma. With the value of NT-proBNP, we can exclude HF in ultrasound-positive pulmonary-related dyspneic patients.

**Table 4:** Test characteristics of ultrasound examination, modified Boston examination, NT-proBNP and combination of ultrasound examination and NT-proBNP^a^

Characteristic	Ultrasound examination^b^	Modified Boston criteria scoring	NT-proBNP	Ultrasound examination + NT-proBNP^c^	*P *value^d^
Sensitivity	100% (95% CI 98 to 100)	85% (95% CI 79 to 89)	92% (95% CI 88 to 95)	100% (95% CI 98 to 100)	< 0.01
Specificity	95% (95% CI 91 to 100)	86% (95% CI 82 to 90)	89% (95% CI 84 to 92)	100% (95% CI 97 to 100)	< 0.01
NPV	100% (95% CI 98 to 100)	80% (95% CI 77 to 85)	86% (95% CI 82 to 90)	100% (95% CI 98 to 100)	< 0.01
PPV	96% (95% CI 93 to 100)	90% (95% CI 86 to 93)	90% (95% CI 85 to 94)	100% (95% CI 96 to 100)	< 0.01
LR^+^	20 (95% CI 1.98 to 89.94)	6.1 (95% CI 1.65 to 18.48)	8.36 (95% CI 1.72 to 33.86)	Infinite	< 0.01
LR^-^	0	0.18 (95% CI 0.07 to 0.52)	0.09 (95% CI 0.02 to 0.23)	0	< 0.01
AUROC	0.94 (95% CI: 0.90 to 0.97)	0.86 (95% CI 0.80 to 0.91)	0.90 (95% CI 0.84 to 0.94)	0.99 (95% CI 0.98 to 1.00)	< 0.01

^a^NPV, negative predictive value; PPV, positive predictive value; LR^+^, positive likelihood ratio; LR^-^, negative likelihood ratio; AUROC, area under receiver-operating curve; NT-proBNP, amino terminal pro-brain natriuretic peptide; UE, ultrasound examination. ^b^UE alone was statistically significantly more accurate in diagnosing HF than the modified Boston criteria and NT-proBNP (better sensitivity, specificity, NPV, PPV, LR^+^, LR^- ^and AUROC; P < 0.01). ^c^The combination of UE and NT-proBNP was the supreme method in diagnosing HF in a prehospital setting; when compared with UE alone, it had equal results in sensitivity, NPV and LR^- ^(P = 0.99) and significantly better results in specificity, PPV and AUROC (P < 0.01). Compared with Boston modified criteria or NT-proBNP alone, UE + NT-proBNP was significantly better with regard to all characteristics (sensitivity, specificity, NPV, PPV, LR^+^, LR^- ^and AUROC; P < 0.01). ^d^The comparison of the four methods was done using the χ^2 ^test with the Bonferroni correction for multiple comparisons. The AUROC accuracy of UE (lung comet-tail sign); NT-proBNP; Boston criteria for diagnosing HF (clinical assessment); and the combination of ultrasound, NT-proBNP and Boston criteria were calculated and compared with univariate Z-score testing. AUROC was compared using the technique proposed by Hanley and Mc Neil [20] and Jannuzzi et al. [21].

The combination of ultrasound examination and NT-proBNP was statistically significantly different from the use of single methods. It had values of 100% sensitivity, 100% specificity, 100% NPV, 100% PPV, LR^+ ^infinite, LR^- ^zero, and AUROC 0.99.

## Discussion

Our study demonstrates that ultrasound examination was the best single method for confirming the diagnosis of acute HF in the prehospital setting. Compared with clinical assessment using modified Boston criteria and NT-proBNP testing, lung ultrasound had a significantly better AUROC with regard to diagnostic accuracy. Furthermore, the combination of ultrasound examination and rapid bedside NT-proBNP testing proved to be an even more reliable method for the identification of acute HF and its differentiation from COPD/asthma-related causes of acute dyspnea.

Acute dyspnea is one of the most common conditions encountered in emergency care settings. Correct diagnosis and treatment are of primary importance, as misdiagnosis can result in deleterious consequences for patients. Timely differentiation of HF from other causes of acute dyspnea (especially in cases of COPD/asthma comorbidity) may be difficult. Physical examination, chest radiography, electrocardiography, and standard biological tests often fail to accurately differentiate HF from pulmonary causes of dyspnea [2,4-6]. Rapid NT-proBNP testing has been confirmed as a highly sensitive and specific biomarker for the diagnosis or exclusion of acute HF in emergency care settings [20,21] and may produce improvements in the prehospital management of patients with dyspnea [7]. The reliability of transthoracic lung ultrasound in differentiating acute dyspnea has been confirmed in some previous studies by Lichtenstein *et al. *[9,10], Cardinale *et al. *[8] and Volpicelli *et al. *[11]. The comet-tail sign (B lines) has been proposed as a simple, non-time-consuming sonographic sign of pulmonary congestion and can be obtained at bedside (also with portable echocardiographic equipment) [22]. Agricolla *et al. *[23] studied the diagnostic accuracy of lung ultrasound in diagnosing intersitial pulmonary edema and found significant positive linear correlations between comet-tail signs and chest radiography, wedge pressure and extravascular lung water quantified by the indicator dilution method. Liteplo *et al. *[12] reported that lung ultrasound could be used alone or could provide additional predictive power to NT-proBNP in the immediate evaluation of dyspneic patients presenting to the emergency department.

The data from our study (similarly to the study by Liteplo *et al. *[12]) suggest that NT-proBNP and ultrasound examinations provide complementary diagnostic information which may be useful in the early evaluation of HF in the prehospital setting (that is, in the field). The combination of these two methods has an excellent statistical value: 100% sensitivity, specificity, NPV and PPV; 99% AUROC; LR^+ ^infinite; and LR^- ^zero. To our knowledge, no previous study has specifically compared the utility of lung ultrasound and NT-proBNP in the out-of-hospital setting, as researchers have focused on the patients in emergency departments and intensive care units. Zechner *et al. *[24] presented two cases of dyspneic patients in whom prehospital lung ultrasound helped to distinguish pulmonary edema from acute exacerbation of COPD and suggested the application of ultrasound in the field.

Prehospital emergency physicians offer the earliest treatment of acute dyspnea, which is performed as soon as clinically possible after the event. On the basis of clinical judgment alone, it is sometimes very difficult to distinguish cardiac from pulmonary causes of dyspnea. If prehospital physicians have the tools of rapid NT-proBNP testing and ultrasound at their disposal, the diagnostic dilemmas in differentiating causes of dyspnea are reduced and the treatment possibilities in clinically obscure cases are mainly improved.

Ultrasound is currently the only imaging method that can be used in the field. It offers an opportunity to extend and improve out-of-hospital diagnostic possibilities and is useful for prehospital emergency physicians with additional knowledge of point-of-care ultrasound diagnostics. Under special circumstances, it may be used by well-educated paramedics [25,26]. The application of the Bedside Lung Ultrasound in Emergency Protocol [27] in the field presents an important moment of transition from in-hospital intensive care medicine to out-of-hospital emergency medicine in the diagnostics and treatment of acute dyspnea. In systems such as Slovenia's, where there are medical doctors in prehospital settings, this methodology could prevent transport and hospitalization. In our next study, we intend to test the efficacy of this methodology for preventing hospitalization and improving cost and time efficiency by using ultrasound in patients with dyspnea. On the basis of the presented data, we have developed a simple algorithm for using ultrasound in patients with dyspnea. If the ultrasound does not show B lines, then the diagnosis is COPD/asthma and further evaluation are unnecessary. If there are B lines, then NT-pro-BNP should be measured. If NT-proBNP is positive, the diagnosis is acute HF, and if NT-proBNP is negative, the diagnosis is COPD/asthma. This algorithm could be a powerful tool for emergency care providers, but further investigation (a larger, multicenter study) is needed to validate the utility of this algorithm in the prehospital setting.

This study has methodological limitations. In our analysis, we included only patients with primary HF or COPD/asthma diagnosed in the field, and this limitation decreases the generalizability of this study to other causes of acute dyspnea in the prehospital setting. The primary aim of our study was to determine the diagnostic accuracy of bedside lung ultrasound and NT-proBNP in differentiating HF-related acute dyspnea from COPD/asthma-related acute dyspnea in prehospital settings.

## Conclusions

Ultrasound examination of the lungs alone or in combination with NT-proBNP testing has high diagnostic accuracy in differentiating acute HF-related from COPD/asthma-related causes of acute dyspnea in prehospital emergency settings. The combination of these two methods helps to improve the diagnostic and treatment possibilities in clinically obscure cases of acute dyspnea in the earliest phases of their appearance. Both methods are simple, non-time-consuming and can be used at bedside or in the field.

## Key messages

• Diagnosis of severe, acute dyspnea in the prehospital arena and/or the emergency department can be challenging, but lung ultrasound is proving to be an accurate new diagnostic tool by itself or in combination with other diagnostic modalities.

• Pulmonary edema gives specific, diffusely vertical artefact line (B lines and comet-tail signs) patterns on ultrasound, unlike the results found in patients with obstructive diseases or pulmonary emboli (generally A lines in both cases).

• The question remains how well specific patterns of diffuse B lines on ultrasound scans correlate with levels of NT-pro-BNP and how they help in making the correct diagnosis.

• In our study, the combination of ultrasound examination and NT-proBNP had 100% sensitivity, 100% specificity, 100% NPV and 100% PPV for differentiating heart failure as the cause of acute dyspnea compared to pulmonary causes in the prehospital setting.

• Both ultrasound examinations and NT-pro-BNP point-of-care assays are quick, accurate and feasible, with high diagnostic accuracy, in the prehospital arena.

## Abbreviations

AUROC: area under the receiver-operating curve; BNP: brain natriuretic peptide; CHF: congestive heart failure; CI: confidence interval; COPD: chronic obstructive pulmonary disease; CVP: central venous pressure; HF: heart failure; LR^+^: positive likelihood ratio; LR^-^: negative likelihood ratio; NPV: negative predictive value; NT-proBNP: N-terminal pro-brain natriuretic peptide; PetCO_2_: partial pressure of end-tidal carbon dioxide; PPV: positive predictive value.

## Competing interests

The authors declare that they have no competing interests.

## Authors' contributions

PG participated in the design of the study and collected and interpreted the data. KP participated in the design of the study, collected the data and wrote a final version of the manuscript. SM collected the data and participated in the coordination of the study. GŠ designed the study, participated in the data collection, performed the statistical analysis and drafted the manuscript. All authors read and approved the final manuscript.
